# The IUCN Green Status of Species: A Call for Mediterranean Botanists to Contribute to This New Ambitious Effort

**DOI:** 10.3390/plants11192592

**Published:** 2022-10-01

**Authors:** Donatella Cogoni, Molly K. Grace, Barney Long, Simone Orsenigo, Giuseppe Fenu

**Affiliations:** 1 Department of Life and Environmental Sciences, University of Cagliari, Via S. Ignazio da Laconi 13, 09123 Cagliari, Italy; 2 Wadham College, University of Oxford, Oxford OX1 3SZ, UK; 3 Re: Wild, P.O. Box 129, Austin, TX 78767, USA; 4 Department of Earth and Environmental Sciences, University of Pavia, 27100 Pavia, Italy

**Keywords:** plant conservation, threatened plants, endemic plants, Mediterranean flora, Red List

## Abstract

In the Mediterranean Basin, a critical focal point for the conservation of plant diversity, there has been a large increase in practical conservation actions for many plant species to prevent extinction and to improve their conservation status; quantifying the effectiveness of these initiatives in reversing species declines is urgently important. In 2021, the International Union for Conservation of Nature (IUCN) launched a new tool that allows the impact of conservation actions on plant species to be assessed. The Green Status of Species is a new set of metrics under the Red List of Threatened Species that assigns species to recovery categories, complementary to the classic extinction risk categories. Crucially, the Green Status of Species provides methods to evaluate the impact of past conservation, and the potential for future conservation impact, on species status and recovery in a standardized way. Considering the efforts made so far for the conservation of Mediterranean threatened plants, using the Green Status of Species would be highly useful to direct future conservation policies. We, therefore, encourage botanists and practitioners working on threatened plants in the Mediterranean area to use this new assessment tool to inform conservation and recovery programs.

## 1. Introduction

The conservation of biodiversity is unanimously considered a priority on a global level, as biological diversity is dramatically declining globally due to numerous severe threats [1,2,3]. To date, despite agreed national and international conservation efforts, there is no evidence that this global loss is decelerating [3,4]. Plants are not an exception: global plant diversity is at risk due to anthropogenic changes in ecosystems, and its conservation is a tremendous global task [5]; current estimates indicate that 39% of plant species are at risk of extinction [6]. Currently available data indicate that the human pressure connected with agriculture, biological resource use, natural system modifications, and residential and commercial development is a key driver of extinction risk in plants worldwide (e.g., [7]). However, the effects of these activities are easily detected; conversely, some well-known threatening factors with less detectable impacts (e.g., climate change, invasive species) are not yet clearly linked to species decline or extinction risk [8,9]. This is possibly due to the lack of knowledge of the ecological processes impacted by such factors, and the lack of reliable risk assessment methods [10,11]. In fact, despite increasing evidence of the impact of climate change on the survival of many species, its impact is quantitatively considered in only a small number of assessments of extinction risk [11,12].

Given this context, the Mediterranean Basin presents a focal area for plant conservation: this region is a priority center of plant diversity, because although it represents only 1.6% of the earth’s surface, it hosts about 7% of the planet’s plant species. For this reason, it has been recognized as one of the world’s plant diversity hotspots [13]. Secondly, the Mediterranean Basin has been the cradle of several of the world’s greatest civilizations which have resulted in anthropogenic modifications to natural habitats for over four millennia, leaving a signature of strong human impact on plant richness, distribution, and dynamics, including extinctions (e.g., [14]). Finally, the basin is one of the regions most susceptible to climate change (e.g., [15,16]). The combination of these three conditions makes the Mediterranean Basin a challenging but critical focal point for the conservation of plant diversity.

One of the main goals of conservation is to avoid extinctions and to reduce rates of species decline. Due to a large amount of threatened taxa relative to the resources available for conservation efforts, an estimation of species’ extinction risk in order to identify taxa and geographical areas that are a priority for conservation is required [17,18]. The IUCN Red List of Threatened Species (global, regional, and national) is widely recognized as the most authoritative information source on the extinction risk of species, because it uses a standardized set of quantitative criteria for assessing species conservation status and threats [19,20]. As a consequence, IUCN Red List criteria are widely used to evaluate the global conservation status of species and to categorize their extinction risk (e.g., [19,21]). The IUCN Red List represents an important starting point for conservation actions and may provide useful information for monitoring changes in the conservation status of species. However, it is essential to underline that the Red List only classifies species by risk status, without establishing direct conservation priorities [17] and while it may suggest conservation needs, it is not the only source of information considered when creating species action plans or policy [8].

## 2. Conservation Activities Carried out So Far

To provide evidence to help guide plant conservation, Red List assessments for certain plant groupings of conservation interest have been undertaken (e.g., [22]). Some initiatives have focused on taxonomic/biological groups (e.g., trees and shrubs, monocots, orchids, etc.; e.g., [23]) and numerous national and subnational Red Lists have been produced (e.g., [24,25,26]). Special attention has been given to assessing endemic plants and to identifying focal areas deserving particular attention for conservation [18]. The results of these assessments have been used to identify those species on the brink of extinction for which conservation is a pressing need, and to inform specific conservation plans, including both in situ and ex-situ measures.

At the same time, sometimes in a poorly coordinated way, there has been a large increase in practical conservation actions for many plant species in the Mediterranean Basin. At first, numerous conservation actions for threatened species, financed by national or international projects, were carried out indirectly through actions designed to benefit umbrella habitats or species. More recently, synergistic conservation actions have been developed; based on a framework that focuses firstly on a precautionary approach (e.g., ex situ germplasm conservation) and subsequently on in situ conservation interventions. In this framework, numerous conservation responses have been implemented to try to halt plant declines: from general large-scale measures, such as the designation of protected areas and international legislation aimed at protecting species, to local species-specific efforts, such as seed-banking programs and conservation translocations. It is well-established that protected areas alone (e.g., Natura 2000 sites, natural parks) do not guarantee per se positive outcomes for species [27,28], so ex situ conservation (e.g., conservation of germplasm in seed banks and cultivation in botanical gardens) is essential and has allowed the conservation of a substantial number of accessions of endangered species throughout the planet (e.g., [29]). To prevent the extinction of threatened plant species and to improve their conservation status, translocations have become increasingly important worldwide (e.g., [30,31]); in particular, conservation translocations are estimated to become very important for conservation in a changing climate [32,33]. Conservation translocations have sharply increased in number in recent decades, as demonstrated by the thousands of translocation projects performed worldwide (e.g., [31,34,35,36,37]), with relevant information available for several threatened plants on regional and national databases (e.g., TRANSLOC; Trans-Planta; IDPlanT [38]). In addition, considering the many limits that remain in the implementation of these conservation actions (e.g., high costs, both economic and time, the availability of suitable translocations sites, access to private areas, the high uncertainty of success principally connected to natural stochastic events, etc.), other active measures have been implemented for numerous threatened plants (e.g., the erection of fences to protect a small population, or cloning the entire population in a local nursery [39,40]).

Given the global increase in practical conservation actions targeting plants, and the accompanying expenditure of resources, quantifying the effectiveness of these initiatives in reversing species declines is important. Being able to quantify and demonstrate the effectiveness of plant conservation is more crucial than ever as conservation programs increasingly need to demonstrate results via evidence and metrics in order to obtain financing [41] and in some cases, funding is provided only once impact is achieved.

In 2021, the International Union for Conservation of Nature (IUCN) launched a new tool that will allow the impact of conservation actions on species to be assessed [42,43,44]. The Green Status of Species is a new set of metrics under the Red List of Threatened Species that assigns species to recovery categories, complementary to the classic extinction risk categories. Crucially, the Green Status of Species provides methods to evaluate the impact of past conservation, and the potential for future conservation impact, on species status and recovery in a standardized way [43,45]. We recommend that the Green Status of Species, along with traditional Red List assessments be used to evaluate the recovery status and conservation impact of Mediterranean Basin plant species.

## 3. The Green Status Approach

The Green Status of Species introduces, and its metrics are based upon, a standardized definition of species recovery. A species is considered Fully Recovered (or Non-Depleted) if it is (1) present, (2) viable (i.e., not threatened with extinction), and (3) ecologically functional, in all parts of its indigenous range (areas that were occupied prior to major human impacts, including areas of natural expansion since that time) [42]. The degree to which a species meets this definition is captured by a *Green Score*, where 100% represents a Fully Recovered or Non-Depleted species while 0% represents a species that is Extinct or Extinct in the Wild. The Green Score at the time of assessment, based on observed or inferred information, is called the *Species Recovery Score*.

However, the assessment does not end there. Relevant to the need for impact evaluation in conservation, a Green Status assessment provides a framework for assessing the impact of conservation actions on the species’ Green Score. In addition to the Species Recovery Score, Green Scores are also estimated under scenarios including or excluding past and expected future conservation actions; assessors provide evidence-based estimates of the Green Score in four specific scenarios [42]. These scenarios are: (1) no past conservation actions occurred; (2) conservation actions continue as planned; (3) all conservation actions are halted; (4) conservation actions are improved and sustained. These scenario-based Green Scores are used to calculate four conservation impact metrics: *Conservation Legacy* (impact of past conservation); *Conservation Dependence* (expected impact of halting all conservation in the short term); *Conservation Gain* (expected impact of continuing conservation in the short term); and *Recovery Potential* (maximum possible recovery within 100 years) [44]. These four metrics are comparable between species and provide new information for use in conservation decision-making.

## 4. Mediterranean Plant Green Status Case Study: *Ribes sardoum* Martelli

One of the first species to be assessed using the Green Status of Species approach was the Sardinian Currant (*Ribes sardoum*) [46]. The Green Status assessment of this plant, which is assessed by the Red List as Critically Endangered [47] demonstrates the value of the Green Status to complement the information in the traditional Red List assessment. The species has a narrow indigenous range, as this endemic has only ever been documented in one location, and further is specialized to limestone habitat; it is inferred that the current location of the species has been its only location since 1750 CE [46]. Within this location, the species is not viable due to ongoing threats from human disturbance and grazing animals. Based on these pieces of information, the calculated Green Score for *Ribes sardoum* is just 33%—far away from the 100% that represents full recovery.

In addition to the current Green Score of *Ribes sardoum*, the Green Status of Species assessment also reports the four additional metrics which measure the impact of past and future conservation action. The species has a Conservation Legacy of 0%, indicating that if past conservation actions (i.e., bolstering the wild population with individuals from ex situ populations and fencing the wild population to reduce grazing pressure) did not occur, the resulting Green Score is predicted to be the same as the current Green Score (in both cases, reflecting a Critically Endangered, but persisting, population). In contrast, the Conservation Dependence, or projected importance of ongoing conservation action, is high. The Conservation Dependence value of 33% indicates that if conservation action stopped today, the species’ Green Score is expected to drop by 33% within the next 10 years; bringing the score to 0% (Extinct in the Wild). The Conservation Gain of the species is 0%, indicating that ongoing and planned conservation actions are not expected to change the Green Score in the next 10 years. Finally, the Recovery Potential indicates that it is still biologically possible for the species’ Green Score to reach 100% (full recovery) within the century. However, this future is uncertain, especially because the single location is vulnerable to extinction via stochastic events, which are expected to increase in frequency with climate change.

## 5. Future Directions

The introduction of the IUCN Green Status of Species as a new tool in the conservation toolbox presents an exciting opportunity to standardize the evaluation of conservation impact and recovery potential between species. However, in order to do this, a dataset of Green Status assessments must first be established; in particular, it would be essential to start evaluating the impact of the many conservation actions carried out so far to guide future conservation measures. Considering the many initiatives/projects carried out and the efforts made for the conservation of plant diversity in the Mediterranean, the ability to evaluate the impact of past conservation, and the potential for future conservation impact, on plant species status and recovery would be helpful in directing conservation actions and policies at the regional or biogeographic level. Given the utility of the new information provided by a Green Status assessment, we encourage scientists and practitioners working on threatened plants in the Mediterranean area to contribute by engaging in Green Status assessments. If you would like to participate, or would like more information, please reach out to the IUCN-SSC Mediterranean Plant Specialist Group via the corresponding author and review the materials on the Green Status website: https://www.iucnredlist.org/resources/green-status-assessment-materials (accessed on 22 September 2022).

## Data Availability

Not applicable.
